# Beer in the United States

**Published:** 1892-11

**Authors:** 


### BEER IN THE UNITED STATES.

The consumption of beer in the United States, exclusive of the large amount imported, is found by the revenue statistics to amount to 31,475,519 barrels in the last year ; or about half a barrel for each man, woman and child in the country. As the actual drinkers can hardly be more than one-fourth of the population, they must swallow an average of two barrels of the stuff, per man. When it is considered that beer is the most pernicious, morally and physically, of all alcoholic beverages, and is more pernicious in our climate and mode of life than elsewhere in the world, these statistics may prepare us to expect any conceivable stages of national degeneracy in the future.

